# Smart pH Sensing: A Self-Sensitivity Programmable Platform with Multi-Functional Charge-Trap-Flash ISFET Technology

**DOI:** 10.3390/s24031017

**Published:** 2024-02-04

**Authors:** Yeong-Ung Kim, Won-Ju Cho

**Affiliations:** Department of Electronic Materials Engineering, Kwangwoon University, Gwangun-ro 20, Nowon-gu, Seoul 01897, Republic of Korea; jobs0506@kw.ac.kr

**Keywords:** pH sensor platform, ion-sensitive field-effect transistor (ISFET), charge trap flash (CTF), self-sensitivity programmability, resistance coupling effects, sensitivity control

## Abstract

This study presents a novel pH sensor platform utilizing charge-trap-flash-type metal oxide semiconductor field-effect transistors (CTF-type MOSFETs) for enhanced sensitivity and self-amplification. Traditional ion-sensitive field-effect transistors (ISFETs) face challenges in commercialization due to low sensitivity at room temperature, known as the Nernst limit. To overcome this limitation, we explore resistive coupling effects and CTF-type MOSFETs, allowing for flexible adjustment of the amplification ratio. The platform adopts a unique approach, employing CTF-type MOSFETs as both transducers and resistors, ensuring efficient sensitivity control. An extended-gate (EG) structure is implemented to enhance cost-effectiveness and increase the overall lifespan of the sensor platform by preventing direct contact between analytes and the transducer. The proposed pH sensor platform demonstrates effective sensitivity control at various amplification ratios. Stability and reliability are validated by investigating non-ideal effects, including hysteresis and drift. The CTF-type MOSFETs’ electrical characteristics, energy band diagrams, and programmable resistance modulation are thoroughly characterized. The results showcase remarkable stability, even under prolonged and repetitive operations, indicating the platform’s potential for accurate pH detection in diverse environments. This study contributes a robust and stable alternative for detecting micro-potential analytes, with promising applications in health management and point-of-care settings.

## 1. Introduction

In recent years, amid global health concerns and increased life expectancies, health management and point-of-care (PoC) applications have garnered significant attention [1,2,3,4]. Reflecting this trend, a fundamental biosensor, the pH sensor, has gained particular attention. Among pH sensors, the ion-sensitive field-effect transistor (ISFET) based on the metal oxide semiconductor field-effect-transistor (MOSFET) structure has been particularly extensively researched. While other pH sensors, such as optical fiber pH sensors, fluorometric pH sensors, and biosensor-based pH sensors, are also under investigation, they have not become primary research topics due to drawbacks such as complexity, high cost, labeling, and the instability of measurement results [5,6,7,8,9,10]. ISFETs stand out with advantages such as accurate sensing operation, label-free detection, compact size, rapid response, and compatibility with complementary metal oxide semiconductor (CMOS) processes, distinguishing itself from these alternatives [11,12,13,14].

However, the commercialization of ISFETs is hindered by its low sensitivity at room temperature (59.14 mV/pH), which is known as the Nernst limit [15,16,17]. Due to the fundamental role of pH sensors, used as diagnostic devices for diseases and environmental pollution, it is crucial to ensure their accurate sensing capabilities for even trace amounts. The Nernst limit is the most significant limitation that hinders precise and high-sensitive detection, which is essential for early problem detection. Addressing this limitation using external amplification circuits results in increased power consumption and complicates the sensor platforms. As an alternative, studies have explored the direct implementation of amplification capabilities in ISFET devices. Notable methods for self-amplification include capacitive and resistive coupling effects [18,19,20,21,22,23].

Capacitive coupling effects leverage the electrical amplification effect between the top- and bottom-gate insulators in a dual-gate (DG) structure. However, this approach poses challenges because the amplification ratio is determined by the dielectric capacitance ratio and cannot be adjusted after fabrication. By contrast, resistive coupling effects utilize the resistance ratio between two resistors to determine the amplification ratio, which facilitates the development of adjustable ratios via the modulation of the resistance of the resistors. The adjustability of the amplification ratio implies that the sensor characteristics can be modified according to the user’s preferences such as power consumption, sensitivity, and stability. This essentially means that a single sensor can effectively adapt to complex scenarios, meeting the user’s desired parameters. A crucial consideration in adjusting the amplification ratio is the selection of devices for the resistors. Adopting charge-trap-flash (CTF) memory devices facilitates the effective implementation of a wide range of amplification ratios through the modulation of the channel resistance changes according to the program or erasure modes [24,25,26].

This study developed a sensor platform using resistive coupling effects and CTF-type MOSFETs, enabling the flexible adjustment of the amplification ratio. An efficient multifunctional system was implemented by employing multiple CTF-type MOSFET devices, each with the same structure as the transducer and resistor. This was realized owing to the unique characteristics of the CTF-type MOSFET used as a transducer, which employed channel resistance modulation owing to charge trapping, similar to a variable resistor [24,25]. Conventional ISFET structures suffer from a vulnerability wherein analytes (pH buffer solution) directly contact the transducer, rendering the entire platform susceptible to contamination and reducing its lifespan. To overcome this, this study adopted an extended-gate (EG) structure to enhance the cost-effectiveness and increase the overall lifespan of the sensor platform [27,28,29]. The pH sensitivity of the proposed sensor platform was evaluated at various amplification ratios to confirm its effective sensitivity control. Furthermore, to validate the stability and reliability of the proposed sensor platform, we investigated non-ideal effects, such as hysteresis and drift effects [30,31,32,33,34].

## 2. Materials and Methods

### 2.1. Fabrication of the Multi-Functional Charge-Trap-Flash-Type Field-Effect Transistor

**Figure 1:**
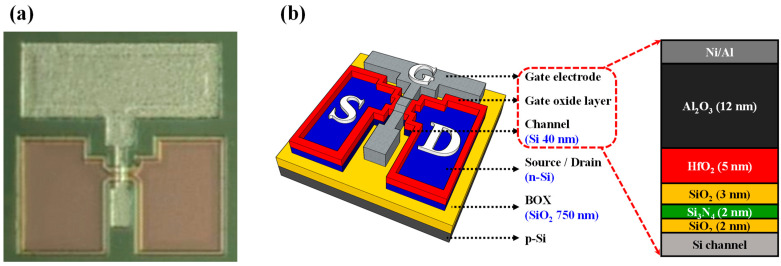
(**a**) Optical microscope image and (**b**) schematic structure with a cross-sectional view of the thin-film layers of the CTF-type MOSFET.

The proposed pH sensor platform relied on a multifunctional CTF-type MOSFET that served as both a transducer and a resistor. The CTF-type MOSFET with a DG structure prepared in this study used p-type (100) bonds and etch-back silicon-on-insulator (BESOI) wafers. The thicknesses of the top-silicon and buried-oxide (BOX) layers was 100 nm and 750 nm, respectively. The top-silicon layer, a MOSFET channel, was a p-type single-crystal silicon with 1 × 10^15^ cm^−3^ of boron doping. The active region of the top-Si layer was defined using photolithography and reactive-ion etching (RIE) with SF_6_ gas. Subsequently, a 100 nm thick heavily phosphorus (n^+^)-doped poly-Si layer was deposited for source/drain (S/D) using low-pressure chemical vapor deposition (LPCVD) at 650 °C. Post-deposition-annealing (PDA) was performed via rapid thermal annealing (RTA) at 850 °C (30 s, N_2_). The n^+^-doped poly-Si layer, except for the S/D region, was completely removed using RIE, and the top-Si layer was etched to a thickness of 40 nm for the channel. As a result, a DG-type nMOSFET with n^+^-doped poly-Si S/D and a thin channel with a 40 nm thickness was fabricated. Surface damage and roughness resulting from the thinning process were carefully eliminated using an ammonia peroxide solution. Channels were defined with widths and lengths of 10 μm each. Before forming the crucial components, a charge-trap layer (CTL), a barrier layer (BL), and a tunneling barrier were established. The tunneling barrier comprised an ONO structure stacked as a 2 nm thick SiO_2_ film (thermal oxidation), a 2 nm thick Si_3_N_4_ film (LPCVD), and a 3 nm thick SiO_2_ film (LPCVD). Subsequently, a 5 nm thick HfO_2_ film for the CTL and a 12 nm thick Al_2_O_3_ film for the BL were deposited using atomic layer deposition (ALD). To enhance the electrical properties of the thin films, PDA was conducted using RTA at 850 °C (30 s, N_2_). Electrodes were formed using a 20/100 nm thick Ni/Al thin film deposited using an electron-beam evaporator. Forming-gas annealing (FGA) at 450 °C (30 min, 2% H_2_/N_2_ mixture) removed any dangling bonds and internal defects in the thin film. Figure 1a shows an optical microscopy image of the fabricated CTF-type MOSFET, and Figure 1b illustrates the schematic structure and cross-sectional view of the thin-film layers.

### 2.2. Characterizations of the Self-Sensitivity Programmable pH Sensor Platform

We assessed the electrical characteristics of the self-sensitive programmable pH sensor platform using an Agilent 4156 B precision semiconductor parameter analyzer (Santa Clara, CA, USA). The device was positioned in a dark box to eliminate optical and electrical interference from the external environment. Pulse application for the channel resistance modulation of the CTF-type MOSFET was achieved using a waveform generator (RIGOL DG900, Suzhou, China). To analyze the pH response, a commercial ceramic plug junction-type Ag/AgCl electrode (Horiba 2080-06T, Kyoto, Japan) with an internal solution of AgCl-saturated KCl electrolyte served as the reference electrode. All measurements were recorded in a dark box in a controlled environment to ensure accuracy. To evaluate the hysteresis and drift effects, we conducted several tests to assess the reliability of the sensor platform under repeated and continuous operations. Hysteresis voltage (*V_H_*) was determined through calculations of the difference in reference voltage (*V_REF_*) between the initial and final pH states in the pH loop of [7→4→7→10→7]. Furthermore, the drift effect was observed by monitoring the shift in the *V_REF_* when exposing the SnO_2_ sensing membrane to a buffer solution of pH 7 for 10 h.

### 2.3. Signal Amplification of the Self-Sensitivity Programmable pH Sensor Platform

**Figure 2:**
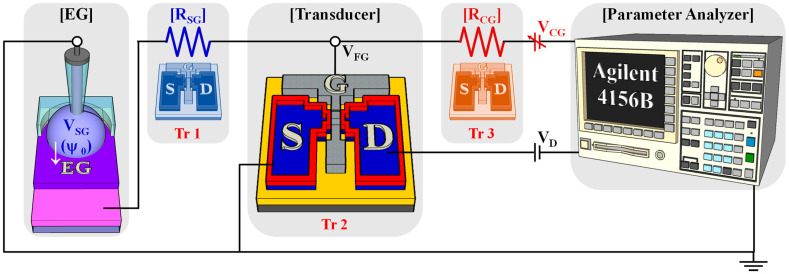
Simplified equivalent circuit for the self-sensitivity programmable pH sensor platform.

The self-sensitivity programmable pH sensor platform demonstrated variable amplification ratios during sensing operations, depending on the resistance ratios of the control gate resistor (*R_CG_*) and sensing gate resistor (*R_SG_*). The sensing gate (SG) comprised an EG, pH buffer solutions, and a reference electrode. This conveyed the surface potential changes owing to the adsorption and desorption of hydrogen ions to and from the transducer. The control gate (CG) was connected to an Agilent 4156B, which supplied the operational voltage to the transducer. In this configuration, *R_CG_*, *R_SG_*, and transducer utilized the same CTF-type MOSFET. Changes were employed in the channel resistance of the device as resistors through pulsed applications. However, the achievement of channel resistance modulation in the CTF-type MOSFET for signal amplification necessitated high voltage (over ∣7∣ V). Consequently, the sensing operation of the transducer, limited to the −5–5 V range, avoided inducing changes in the channel resistance, enabling the implementation of a multifunctional device. The equivalent circuit of the proposed self-sensitivity-programmable pH sensor platform is shown in Figure 2. The gate voltage (*V_G_*) of ISFETs is expressed as Equation (1). The voltage between CG and SG is expressed as Equation (2). A change in the potential of SG (*Δψ_0_*) was amplified by the resistive coupling ratio (*R_CG_*/*R_SG_*), causing a change in *V_CG_*. This implied that even micro-potential analytes could be detected with high sensitivity via resistive coupling [18,26].

(1) $$V_{FG}=\left(\frac{R_{SG}}{R_{SG}+R_{CG}}\right)V_{CG}+\left(\frac{R_{CG}}{R_{SG}+R_{CG}}\right)V_{SG}$$

(2) $$V_{CG}=\left(\frac{R_{SG}+R_{CG}}{R_{SG}}\right)V_{FG}-\frac{R_{CG}}{R_{SG}}V_{SG}$$

(3) $$∴{∆V}_{CG}\propto \;\frac{R_{CG}}{R_{SG}}{∆V}_{SG}$$

## 3. Results and Discussion

### 3.1. Evaluation of the Multi-Functional Charge-Trap-Flash-Type Field-Effect Transistor

**Figure 3:**
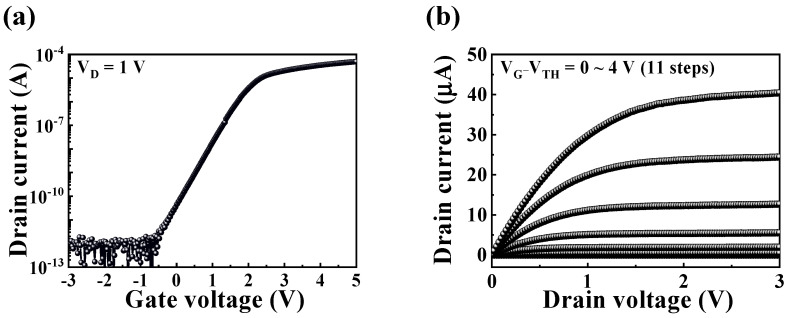
Electrical characteristics of CTF-type MOSFET: (**a**) transfer curve, and (**b**) output curve.

Figure 3a,b show the typical electrical characteristics of the transfer (*I_D_*–*V_G_*) and output (*I_D_*–*V_D_*) curves, respectively. As shown in Figure 3a, the drain current (*I_D_*) was recorded at a constant drain voltage (*V_D_*) of 1 V while applying a *V_G_* ranging from −3 to 5 V. The threshold voltage (*V_TH_*) was determined via linear extrapolation of the *I_D_*–*V_G_* curve in the linear region. Figure 3b illustrates *I_D_* as *V_G_*–*V_TH_* changed from 0 to 4 V across 11 steps. The current exhibited a linear increase in the low *V_D_* region and subsequently pinched off as *V_D_* increases, resulting in saturation characteristics.

The electrical parameters of the multi-functional CTF-type MOSFET were derived using the following equations [35,36]

(4) $$SS={\left(\frac{dlogI_{D}}{dV_{G}}\right)}^{-1}$$

and

(5) $$\mu_{FE}=\left(\frac{Lg_{m}}{W\cdot C_{ox}\cdot V_{D}}\right),\;g_{m}=\frac{{\partial I}_{D}}{{\partial V}_{G}}$$

where *W* is the channel width, *L* is the channel length, *C_ox_* is the capacitance per unit area of the gate oxide, and *g_m_* is the transconductance. The values extracted for various electrical parameters from the multi-functional CTF-type MOSFET were as follows: *V_TH_* was approximately −0.25 V, the on/off current ratio (*I_on_*/*I_off_*) was 1.49 × 10^7^, the field-effect mobility (*μ_FE_*) was 234.92 cm^2^/V·s, and the subthreshold swing (*SS*) was 234.92 mV/dec. Table 1 summarizes the electrical parameters.

**Figure 4:**
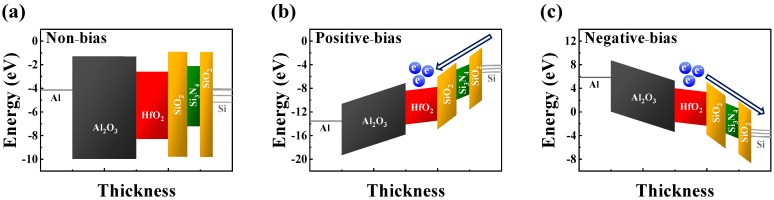
Energy band diagram of CTF-type MOSFET under (**a**) non-bias, (**b**) positive-bias, and (**c**) negative-bias states.

Figure 4a,b show the energy band diagrams of the thin-film layer of the MAHONOS stack under non-, positive-, and negative-gate-bias conditions, respectively [37,38,39]. In these diagrams, the Al_2_O_3_ layer functioned as a BL, offering a high dielectric constant, significantly large bandgap offset, and substantial physical oxide thickness (POT) [40,41,42]. The selection of the HfO_2_ layer as the CTL was motivated by its higher trap density, greater dielectric constant, and lower bandgap offset compared with Al_2_O_3_ or SiO_2_ layers [43,44,45,46,47]. The engineered ONO structure, referred to as the variable oxide thickness (VARIOT) tunnel barrier, exhibited notable sensitivity to the electric field generated by the gate bias [48,49]. When the energy level of the electrons within the silicon channel was lower than that of the potential barrier, the ONO barrier functioned as an obstacle to electron penetration [50,51,52,53]. However, a substantial electric field induced significant band bending within the ONO barrier, allowing the electron wavefunction to tunnel through the thin triangular potential barrier. Consequently, the channel conductance in the CTL was modulated through charge trapping (leading to decreased conductance) or de-trapping (leading to increased conductance). In the retention mode, the trapped charges remained stable in the CTL, and the charge loss rate decreased owing to the substantial POT of the ONO barrier [54,55,56].

**Figure 5:**
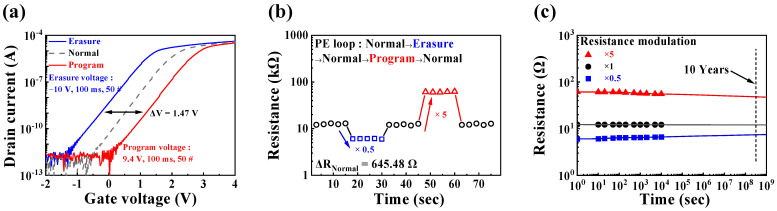
(**a**) Transfer curves, (**b**) hysteresis resistance, and (**c**) retention time according to the program/erasure modes.

Figure 5 shows the transfer curves (a), hysteresis resistance (b), and retention time (c) corresponding to the program/erasure mode of the CTF-type MOSFET. We define the normal state of the CTF-type MOSFET as the state without an external voltage, the state after applying a positive bias as the program state, and the state after applying a negative bias as the erasure state. The changes in the electrical characteristics were measured for each state [57,58,59].

In the program mode, 50 pulses of 9.4 V with a duration of 100 ms were applied, whereas −10 V was utilized in the erasure mode. Depending on the magnitude and type of voltage applied to the gate, a *V_TH_* shift of approximately 1.47 V occurred. This shift was attributed to charge trapping and de-trapping, induced by the voltage applied to the top gate [60,61,62].

Hysteresis resistance was observed by cycling through a [normal–program–normal–erasure–normal] operation loop, and the change in the channel resistance after the loop was measured by expressing the difference between the initial and final values as *ΔR_normal_*. The measured value of *ΔR_normal_* was 645.48 Ω, representing approximately 5.33% of the resistance value in the normal state.

The retention time, which indicates how long the changed channel resistance is maintained for after the program/erasure mode, was also investigated [63,64,65]. Through the analysis of the records measured over 10,000 s, the change in resistance after 10 years was estimated. Percentage changes were observed as +22.28% at ×0.5, −2.02% at ×1, and −21.41% at ×5.

The measured results confirmed that the channel resistance modulation of the CTF-type MOSFET demonstrated significant stability, even under long-term and repetitive conditions. This indicates that there are no issues with its operation as a resistor in the proposed self-sensitivity programmable pH sensor platform.

**Figure 6:**
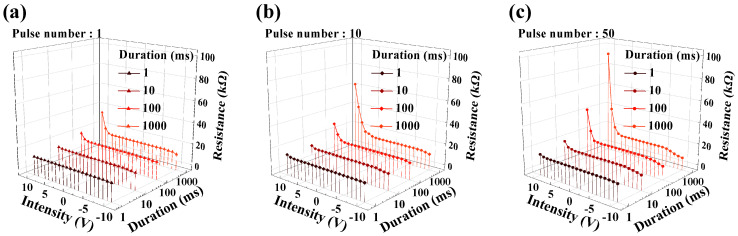
Channel resistance modulation with input pulse application. Conditions include the number of pulses: (**a**) 1, (**b**) 10, and (**c**) 50.

Figure 6 depicts the channel resistance modulation of CTF-type MOSFET under various input voltage conditions. To construct a self-sensitive programmable pH sensor platform, it is crucial to accurately determine the *R_CG_* and *R_SG_* values for amplification. Therefore, this study investigated the channel resistance modulation according to the applied voltage type of the CTF-type MOSFET used as a resistor.

The objective of this experiment was to broadly configure the channel resistance variation based on the program/erase operation of the memory device, allowing the implementation of various amplification ratios. To secure various channel resistance modulation conditions, pulse-type program/erase voltages were applied instead of constant voltage. The pulse conditions used for measurements included pulse numbers of 1–50, pulse amplitudes of −10–10 V, and durations of 1–1000 ms. Resistance extraction was conducted for a total of 252 conditions, and the extracted resistance values ranged from 6.62 to 91.55 kΩ based on the measurement results. Therefore, it was confirmed that sensitivity adjustment is possible, ranging from a minimum of ×0.07 to a maximum of ×13.83 when combining resistance ratios. This signifies the successful fulfillment of the minimum conditions for constructing a self-sensitivity programmable pH sensor platform. Based on these results, subsequent measurements were implemented, with the amplification ratio specified as ×0.5, ×1, and ×5.

### 3.2. pH Sensing Characteristics of the Self-Sensitivity Programmable pH Sensor Platform

**Figure 7:**
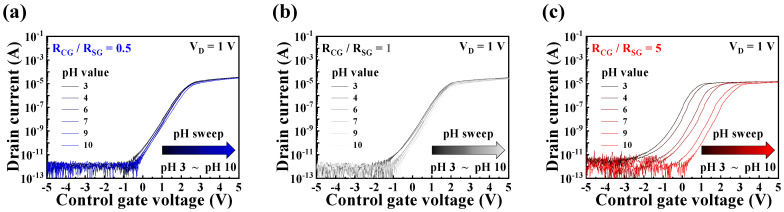
pH sensing operation of self-sensitivity programmable pH sensor with amplification ratio of (**a**) ×0.5, (**b**) ×1, and (**c**) ×5.

To evaluate the pH detection and signal amplification capabilities of the proposed pH sensor platform, we established the amplification ratio and examined the changes in the transfer curve as a function of CG. Figure 7 shows the alterations in the transfer curve when the amplification ratios were set to ×0.5, ×1, and ×5. The transfer curve was recorded in the range of −5–5 V using a buffer solution with pH values of 3, 4, 6, 7, 9, and 10. The sensing operation range was determined based on the conditions verified in Figure 6, setting it to the range (∣V∣ < 7 V) where the channel resistance of the transducer unit, the CTF-type MOSFET, remained unchanged.

The pH buffer solution that came into contact with the EG sensing unit caused a change in surface potential through ion adsorption on the sensing membrane’s surface. This change was then transmitted through the EG electrode to the top gate of the CTF-type MOSFET. In this process, *ΔV_SG_* was amplified in proportion to the ratio of *R_CG_*/*R_SG_*, leading to variations in the *V_TH_* shift of the transfer curves. As observed in Figure 7, surface potential changes due to pH sensing occur more prominently with increasing amplification ratios, resulting in a noticeable shift in the transfer curve. Consequently, the variation in *V_REF_* is determined accordingly.

**Figure 8:**
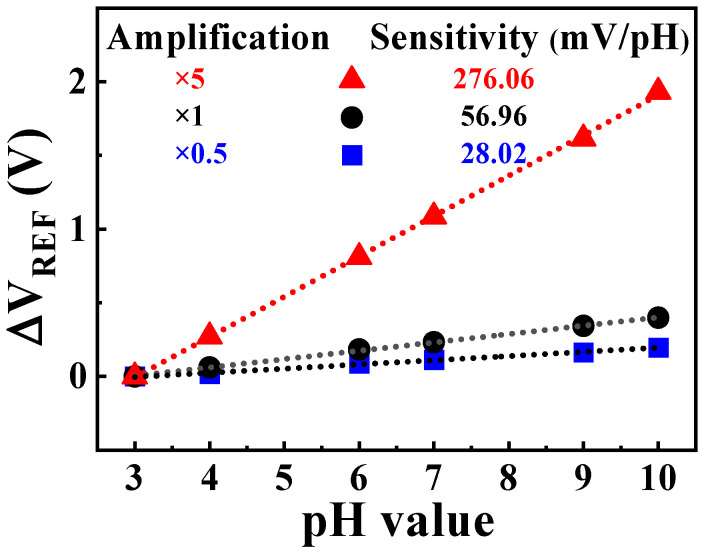
Programmed pH sensitivity values based on the amplification ratio.

Figure 8 shows the sensitivity data extracted from each transfer curve. The amplification ratio was determined via adjustments to the *R_CG_*/*R_SG_* value, resulting in pH sensitivities of ×0.49 amplification (28.02 mV/pH), ×1 amplification (56.96 mV/pH), and ×4.85 amplification (276.06 mV/pH). Also, the linearity of each sensitivity value was 98.51% at ×0.49 amplification, 98.88% at ×1 amplification, and 98.95% at ×4.85 amplification, respectively.

The measurement results demonstrate that the proposed self-sensitivity programmable pH sensor platform achieved sensitivities close to the configured amplification ratios while also ensuring excellent linearity for pH buffer solutions with different values. This indicates the successful and stable implementation of the intended sensitivity programming capability.

**Figure 9:**
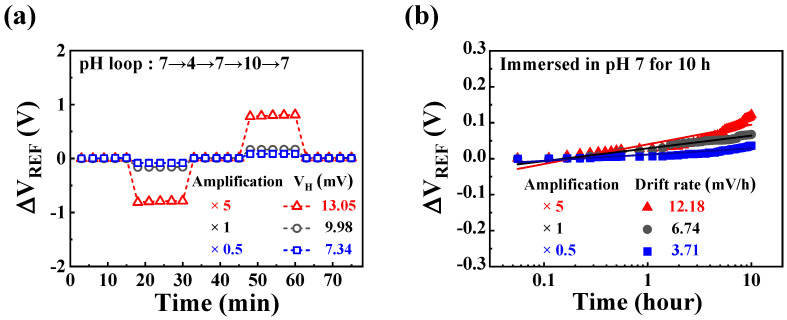
Non-ideal effect of the self-sensitivity programmable pH sensor platform: (**a**) hysteresis voltage, and (**b**) drift rate.

To assess the stability of the proposed pH sensor platform during repeated and continuous operation, we evaluated the non-ideal effects, including hysteresis and drift [66,67,68]. In Figure 9a, the hysteresis voltage is presented after a pH loop [7→10→7→4→7]. This was evaluated to estimate the stability in environments where different analytes needed to be detected repeatedly. The measurements were carried out at 2 min intervals for a total of 35 cycles in accordance with the specified pH loop, defining the hysteresis voltage as the difference between the initial and final values. The measured results showed values of 7.34, 9.98, and 13.05 mV for amplification ratios of ×0.5, ×1, and ×5, respectively. Figure 9b illustrates the drift effect measured after 10 h of exposure to a pH 7 buffer solution, evaluated in relation to stability when measuring a specific analyte over an extended period. The drift rate was expressed as a function of time, representing the difference between the initial and final values after 10 h of measurement. The measured results showed values of 3.71, 6.74, and 12.18 mV/h for amplification ratios of ×0.5, ×1, and ×5, respectively.

In both hysteresis voltage and drift rate, the measured results showed an increasing trend in non-ideal effect values with the rising amplification ratio. However, since the rate of increase in non-ideal effect values was considerably lower than the increase in sensitivity, it was evident that the benefits of increased sensitivity through amplification outweighed the negative impact of increased non-ideal effects through amplification. These results indicate that the proposed self-sensitivity programmable pH sensor platform demonstrates excellent stability. As the amplification ratio increases, the platform effectively withstands non-ideal effects, even in a continuous and repetitive measurement environment.

**Figure 10:**
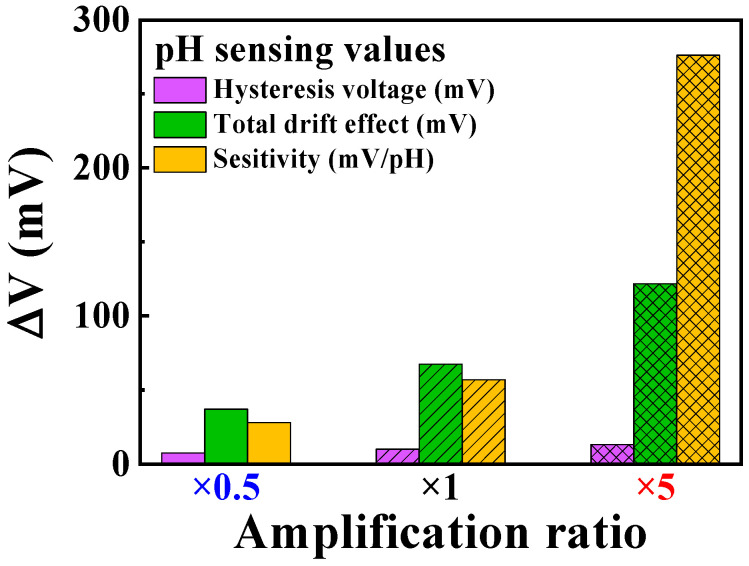
Comparative analysis of sensitivity and non-ideal effects in pH sensing operations.

Table 2 summarizes the observed non-ideal effects and sensitivity values of the self-sensitivity programmable pH sensor platform based on the amplification ratios. As the amplification ratio increases, the numerical values of non-ideal effects also increase, seemingly indicating a decrease in the stability of the sensor platform. However, considering the increase in sensitivity, it can be noted that stability actually improves. Comparative data to confirm this are presented in Figure 10.

Figure 10 shows a quantitative comparison between the sensitivity and non-ideal effects in the pH-sensing operation. The parameters compared were sensitivity (*ΔV_REF_* per unit pH), total drift rate, and hysteresis voltage. Without amplification, ISFETs’ low sensitivity poses challenges as certain non-ideal effect values become larger than *ΔV_REF_*. This issue intensifies during prolonged measurements, rendering it difficult to discern whether *ΔV_REF_* variation is owing to pH differences or sensor platform instability. Consequently, accurate detection is impossible. Conversely, with the applied amplification, relatively accurate measurements are achievable owing to the significantly larger sensitivity values compared to the non-ideal effect values. This was quantitatively confirmed. The ratios of the hysteresis voltage to sensitivity were 26.19% at ×0.5 amplification, 17.52% at ×1 amplification, and 4.73% at ×5 amplification. Similarly, the ratios of the total drift effect to the sensitivity were 132.41% at ×0.5 amplification, 118.33% at ×1 amplification, and 44.12% at ×5 amplification, respectively.

The results demonstrated that, even after repetitive measurements of various pH values and continuous measurements for 10 h, the amplified sensitivity to unit pH was significantly larger than the total non-ideal effects. This indicates that the proposed sensor platform, with applied amplification, could accurately determine the pH of analytes, even in harsh environments. Therefore, the amplification ensured accurate and stable operational characteristics.

## 4. Conclusions

This study presents a novel pH sensor platform that leverages charge-trap-flash-type metal oxide semiconductor field-effect transistors (CTF-type MOSFETs) for enhanced sensitivity and self-amplification. Traditional ion-sensitive field-effect transistors (ISFETs) face challenges with commercialization due to low sensitivity at room temperature, known as the Nernst limit. To overcome this limitation, the research explores resistive coupling effects and CTF-type MOSFETs, enabling the flexible adjustment of the amplification ratio. The platform’s unique approach utilizes CTF-type MOSFETs as both transducers and resistors, allowing for efficient sensitivity control. To implement this, the electrical characteristics, energy band diagrams, and programmable resistance modulation of the CTF-type MOSFET are thoroughly characterized.

An extended-gate (EG) structure enhances cost-effectiveness and increases the sensor platform’s overall lifespan by preventing direct contact between analytes and the transducer. The proposed pH sensor platform demonstrates effective sensitivity control at various amplification ratios, showcasing stability and reliability even under repetitive and continuous operations. The key achievements of the study include the successful implementation of resistive coupling effects and CTF-type MOSFETs, enabling a multifunctional system with programmable sensitivity.

The results of sensitivity measurements demonstrate that the proposed self-sensitivity programmable pH sensor platform achieves amplification ratios very close to the intended ×0.5, ×1, ×5, with achieved amplification factors of approximately ×0.49 (28.02 mV/pH), ×1 (56.96 mV/pH), and ×4.85 (276.06 mV/pH), respectively. Further, the proposed sensor platform exhibits remarkable stability and reliability, as validated by the investigation of non-ideal effects such as hysteresis and drift.

The findings of this research contribute a robust and stable alternative for detecting micro-potential analytes, with promising applications in health management and point-of-care (PoC) settings. The ability to adjust sensitivity programmatically enhances the platform’s versatility, making it suitable for diverse environments and applications. Overall, the proposed pH sensor platform represents a significant advancement in the field of biosensors, paving the way for improved and reliable pH detection methodologies.

## Figures and Tables

**Table 1:** Electrical parameters of the multi-functional CTF-type MOSFET.

*V_TH_* (V)	*SS* (mV/dec)	*μ_FE_* (cm^2^/V·s)	*I_on_/I_off_* (A/A)
−0.25	175.54	234.92	1.49 × 10^7^

**Table 2:** Summarization of non-ideal effects and sensitivity values of the self-sensitivity programmable pH sensor platform according to the amplification ratio.

Amplification Ratio	Hysteresis Voltage (mV)	Drift Rate (mV/h)	Sensitivity (mV/pH)
×0.5	7.34	3.71	28.02
×1	9.98	6.74	56.96
×5	13.05	12.18	276.06

## Data Availability

Data are contained within the article.
